# Searching for mutations in the *HNF1B* gene in a Brazilian cohort with renal cysts and hyperglycemia

**DOI:** 10.20945/2359-3997000000138

**Published:** 2019-04-26

**Authors:** Renata P. Dotto, Lucas Santos de Santana, Susan C. Lindsey, Lilian Araújo Caetano, Luciana F. Franco, Regina Célia M. S. Moisés, João R. Sa, José Luiz Nishiura, Milena Gurgel Teles, Ita P. Heilberg, Magnus R. Dias-da-Silva, Fernando M. A. Giuffrida, André F. Reis

**Affiliations:** 1 Universidade Federal de São Paulo Disciplina de Endocrinologia Departamento de Medicina Universidade Federal de São Paulo São Paulo SP Brasil Disciplina de Endocrinologia, Departamento de Medicina, Universidade Federal de São Paulo (Unifesp), São Paulo, SP, Brasil; 2 Universidade de São Paulo Unidade de Endocrinologia Genética/LIM25 Faculdade de Medicina Universidade de São Paulo São Paulo SP Brasil Grupo de Diabetes Monogênico, Unidade de Endocrinologia Genética/LIM25, Faculdade de Medicina, Universidade de São Paulo (FMUSP), São Paulo, SP, Brasil; 3 Universidade Federal de São Paulo Departamento de Medicina Universidade Federal de São Paulo São Paulo SP Brasil Disciplina de Nefrologia, Departamento de Medicina, Universidade Federal de São Paulo (Unifesp), São Paulo, SP, Brasil; 4 Universidade do Estado da Bahia Departamento de Ciências da Vida Universidade do Estado da Bahia Salvador BA Brasil Departamento de Ciências da Vida, Universidade do Estado da Bahia (UNEB), Salvador, BA, Brasil

**Keywords:** HNF1B, MODY, monogenic diabetes, diabetes

## Abstract

**Objective:**

To verify the presence of variants in *HNF1B* in a sample of the Brazilian population selected according to the presence of renal cysts associated with hyperglycemia.

**Subjects and methods:**

We evaluated 28 unrelated patients with clinical suspicion of *HNF1B* mutation because of the concomitant presence of diabetes mellitus (DM) or prediabetes and renal cysts. Genotyping was accomplished using Sanger sequencing or multiplex ligation-dependent probe amplification (MLPA). In positive cases, available relatives were recruited.

**Results:**

We found two patients with *HNF1B* mutations. The first presented the variant p.Pro328Leufs*48(c.983delC) and had DM, renal cysts, and hypomagnesemia. The second presented a heterozygous whole gene deletion in *HNF1B,* DM, renal cysts, body and tail pancreatic agenesis, and hypomagnesemia; this alteration was also found in his two siblings and his father.

**Conclusion:**

The recruitment of suspected cases of *HNF1B* gene mutations in Brazilians due to hyperglycemia and renal cysts presents two positive cases. Our cases contribute to the annotation of clinical and biochemical phenotypes of this rare form of maturity-onset diabetes of the young (MODY).

## INTRODUCTION

Maturity-onset diabetes of the young (MODY) is characterized by the occurrence of early-onset diabetes mellitus (DM), most often before age 25, with dominant autosomal inheritance caused by primary defects in insulin secretion. MODY is currently known to comprise a group of diseases caused by at least 14 different genetic etiologies. In rarer forms, identification of additional families demonstrating the co-segregation of the genetic variation with DM is necessary to confirm that they may be effectively regarded as MODY genes ( 1 , 2 ).

The *HNF1B* gene was associated with cystic kidney disease shortly after being first described as a cause of MODY by Horikawa and cols. ( 3 ). This disease was initially called renal cysts and diabetes (RCAD) syndrome ( 4 ). However, with the growth in the number of published cases, its broad clinical heterogeneity has been increasingly recognized. It is noteworthy that not all patients carrying an *HNF1B* mutation have renal cysts or DM ( 5 ). Given that *HNF1B* is expressed in many organs, predominantly in the liver, intestines, kidneys, pancreas, and urogenital tract, phenotypes in these organs are the most common and best described ( 6 ). Due to the great clinical heterogeneity among affected patients, various recruitment strategies for patients with suspected *HNF1B* mutations have been proposed with varying accuracy levels ( 7 - 9 ).

The objective of our study was to verify the presence of variants in the *HNF1B* gene in a sample of the Brazilian population selected according to the presence of renal cysts and hyperglycemia.

## SUBJECTS AND METHODS

### Subjects

We recruited twenty-eight patients with clinical suspicion of *HNF1B* gene mutations from the Nephrology and Diabetes outpatient clinics at *Universidade Federal de São Paulo* (Unifesp), and the Diabetes Unit at *Universidade de São Paulo* (USP). These patients presented either DM or prediabetes as per American Diabetes Association criteria ( 10 ) and renal cysts identified by ultrasound or computed tomography (CT) scans during routine medical care. Nineteen patients had autosomal dominant polycystic kidney disease (ADPKD) based on clinical phenotype, defined by number of renal cysts according to age and one affected parent ( 11 ), whereas the other nine had simple renal cysts.

The clinical and biochemical features of these patients were collected from their medical records. In the case of patients with identified *HNF1B* variants, relatives were invited to participate in the study. Patients with type 1 DM, i.e., those with one or more positive autoantibodies against GAD, IA2, or insulin (measured only in individuals without insulin use) and undetectable fasting C-peptide levels, were excluded. Patients were classified as having arterial hypertension and/or dyslipidemia ( 12 ).

After defining the results with the completed genotypes, we were interested in verifying the accuracy of the Faguer score in our sample. In brief, in this paper’s authors devised a score using a weighted combination of the most discriminative clinical, laboratory, and radiological characteristics of patients from published literature ( 13 ).

The study was approved by Hospital São Paulo’s ethics committee, and all patients provided informed consent.

### Genetic analyses

Genomic DNA was extracted from peripheral blood using an in-house method ( 14 ). Next, genetic analyses were performed in two steps: 1) *multiplex ligation dependent probe amplification* (MLPA) and 2) Sanger sequencing.

MLPA was performed using SALSA^®^ MLPA^®^ Probemix P241-E1 MODY Mix 1 with 4 healthy controls in each run. We then analyzed the data using the Coffalyser^TM^ software, following the manufacturer’s protocol and performing intra- and intersample normalization.

Analysis of the 9 exons of *HNF1B* and the promoter region was performed by DNA amplification through polymerase chain reaction followed by Sanger sequencing using a Big Dye Terminator^TM^ Cycle Sequencing Ready Reaction Kit and ABI PRISM 3130 *xl* Genetic Analyzer (Applied Biosystems, Foster City, CA, USA), as previously described ( 15 ). Primers used are detailed in Supplementary Table 1 . Cloning experiments to confirm the mutations were performed using a TOPO TA Cloning^TM^ Kit for Sequencing (Invitrogen/Thermo Scientific^TM^, Carlsbad, CA, USA) in accordance with the manufacturer’s instructions. Plasmid DNA was then extracted using PureLink Quick Plasmid MiniPrep kits (Invitrogen/Thermo Scientific, Grand Island, NY, USA) or QIAfilter Plasmid Maxi (QIAgen^TM^, Hilden, Germany) and submitted to direct sequencing as described above.

**Table 1:** Main clinical and laboratory characteristics of studied patients (n = 28)

Age (yrs)	52.6 ± 12.1
DM/prediabetes (%)^a^	38/62
IMC (kg/m^2^)	27.7 ± 6.5
Fasting glucose (mg/dL)	129.5 ± 38
HbA1c (%)^b^	7 ± 2
HbA1c (mmol/mol)^b^	53.9 ± 22.1
Creatinine (mg/dL)	1.3 ± 0.5
eGFR (mL/min)	67.6 ± 27
Magnesium (mg/dL)	1.8 ± 0.4
Uric Acid (mg/dL)	6.3 ± 1.6
Hypertension (%)	92
Dyslipidemia (%)	7

^a^ DM and prediabetes according to ADA criteria; ^b^ HbA1c measured by HPLC. Continuous variables expressed in mean+SD.

### Statistical analysis

Continuous variables were expressed in mean ± SD. Dichotomous variables were described as percentages.

## RESULTS

Clinical and biochemical characteristics of recruited patients are described in Table 1 .

**Figure 1:**
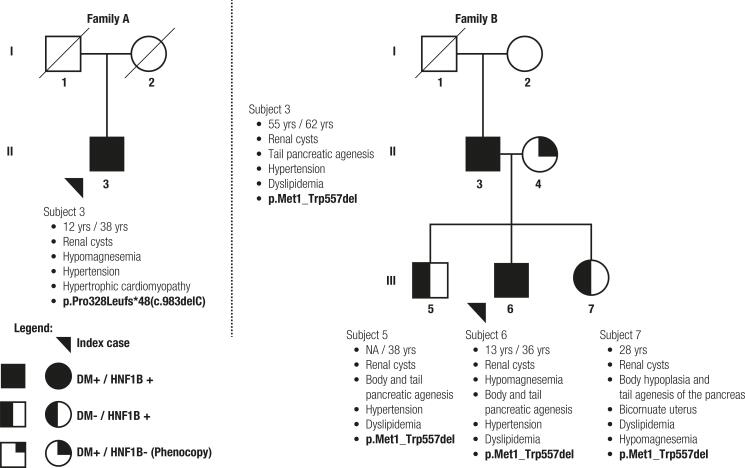
Pedigrees of families with alterations in the HNF1B gene. The two index cases described in the main text are indicated by arrows in the figure. Genotype and diabetes phenotype are indicated as described in the legend box in the figure. Text bullets alongside each of the described individuals show subject number in the pedigree with initials, age at diagnosis of diabetes/current age, associated phenotypes, and mutation found in each individual.

Twenty-eight patients with DM or prediabetes and renal cysts were studied. Six (21%) were on insulin therapy and 7 (25%) were on oral antidiabetic drugs. Twenty-six patients had hypertension (92%), 2 (7%) had dyslipidemia, and 2 (7%) had hypomagnesemia. We found two patients carrying *HNF1B* variants ( Figure 1 and Table 2 ). In addition, a few intronic SNPs and a few variants in the promoter region were found, but they present a high minor allele frequency, indicating that they are variants commonly found in the general population and, thus, do not suggest a relevant impact.

**Table 2:** Individual clinical and laboratory characteristics of patients who presented with HNF1B alterations

Patient	Age/Age at diagnosis (y)	BMI (kg/m^2^)	Fasting glucose (mg/dL)	HbA1c (%)/ mmol/mol	Urea / creatinine (mg/dL)	eGFR (mL/min)^a^	Magnesium (mg/dL)	Uric Acid (mg/dL)	Fasting C-Peptide (ng/mL)	GAD / IA2 antibodies	Family history	Comorbidities	Faguer Score	Imaging exams^b^	Current treatment DM
A-3 (index)	38/12	19.4	165	13.2 / 121	43 / 1.2	76.2	1	NA	0.9	Negative	No DM	Hypertension, Hypertrophic cardiomyopathy, hypomagnesemia	14	Bilateral renal cysts	NPH insulin
B-6 (index)	36/13	22.9	231	7.1 / 54	60 / 1.17	92.2	0.9	5.9	1.4	Negative	DM	Exocrine pancreatic insufficiency; hypertension; Dyslipidemia; Subclinical hypothyroidism (positive thyroperoxidase antibodies) Hypomagnesemia	14	Agenesis of body and tail of pancreas; Bilateral renal cortical cysts	NPH + Regular Insulin
B-3 (father)	62/55	22.7	148	9.5/ 80	39 / 1.22	73	1.7	5.6	2,6	Negative	DM	Hypertension, Dyslipidemia	8	Agenesis of the pancreatic tail; Peripyelic cyst in the middle third of the right kidney	NPH and Regular Insulin + Gliclazide
B-5 (brother)	38/NA	25.2	88	5.1/32	56 / 1.89	50.9	1.42	11.1	3	Negative	DM	Hypertension, Dyslipidemia	12	Agenesis of body and tail of pancreas; Bilateral renal cysts	NA
B-7	28/NA	19.5	88	4.3 /23	21 / 0.66	139	0.9	2.9	1.9	Negative	DM	Dyslipidemia Hypomagnesemia	18	Body hypoplasia and agenesis of the tail of the pancreas; Bilateral renal cortical cysts; Bicornuate uterus	NA

^a^ eGFR (mL/min): methods CKD-EPI; ^b^ Imaging exams: ultrasound or tomography; NA: not applicable.

The first patient with an *HNF1B* variant was a 38-year old male with a BMI of 19.4 (kg/m^2^) who was hospitalized due to headache. He was diagnosed with DM at age 12, had been using NPH insulin since one year after diagnosis, and had arterial hypertension. He presented with a fasting glucose of 165 mg/dL/9.2 mmol/L, HbA1c 13.2%/121 mmol/mol, serum creatinine 1.2 mg/dL, estimated Glomerular Filtration Rate (eGFR) 76.2 mL/min, and magnesium 1.0 mg/dL (normal range: 1.7-2.6 mg/dL). He had negative pancreatic autoantibodies (GAD, IA 2 , and anti-insulin) and fasting C-peptide of 0.9 ng/mL. He carried *HNF1B* variant p.Pro328Leufs*48 (c.983delC), which was identified by Sanger sequence and cloning. This variant was described by Clissold and cols. ( 16 ). There were no available relatives for investigation (both parents had died from unknown causes), but the patient denied any familial history of DM or renal cysts.

The other case was a 36-year old male with a BMI of 22.9 (kg/m^2^) in whom MLPA demonstrated heterozygous whole gene deletion of *HNF1B* (p.Met1_Trp557del). He had had DM since age 13 and presented with a fasting glucose of 231 mg/dL /12.8 mmol/L, HbA1c 7.1%/54 mmol/mol, serum creatinine 1.17 mg/dL, eGFR 92.2 mL/min, and magnesium 0.9 mg/dL. He had been using NPH insulin and regular insulin. He had negative antibodies against GAD and IA2 and fasting C-peptide of 1.4 ng/mL. Imaging studies demonstrated body and tail pancreatic agenesis. The same mutation was identified in his father, who had DM, renal cysts, and agenesis of the pancreatic tail, and in his two normoglycemic siblings, one brother with body and tail pancreatic agenesis and renal cysts and one sister with pancreatic body hypoplasia and tail agenesis, renal cysts, and bicornuate uterus. These cases illustrate the clinical heterogeneity of *HNF1B* phenotypes that can be seen even within the same family.

After analyzing the whole sample, we applied the score devised by Faguer and cols. All 5 positive cases (including all studied family members) had a minimum score of 8 points, which is suggested by the above cited reference as the optimal cutoff threshold for the negative predictive value to rule out *HNF1B* mutations in a suspected individual ( Table 2 ).

## DISCUSSION

To the best of our knowledge, this is the first systematic investigation of *HNF1B* variants in Brazilians with hyperglycemia and renal cysts, phenotypes usually seen in patients carrying variants in this gene ( 17 ). We detected 2 out of 28 cases (7%). Previously, we described an isolated case of *HNF1B* mutation in a Brazilian patient with familial DM and hypomagnesemia recruited due to suspicion of MODY ( 15 ).

The prevalence of mutations in *HNF1B* is variable, mainly due to the high degree of clinical heterogeneity and various recruitment strategies regarding age range and distinct phenotypes. Kanthimathi and cols. studied 50 Indian patients selected according to the presence of renal disease (such as renal cysts, focal segmental glomerulosclerosis, or tubulointerstitial nephritis) and DM and found *HNF1B* variants in 6 individuals (12%) ( 9 ). Horikawa and cols. studied a total of 230 non-obese Japanese patients aged ≤ 35 years with negative autoantibodies against beta cells and found 6 with mutations in *HNF1B* (2.6%) ( 18 ). Edghill and cols. studied 133 patients from the UK with renal disease of unknown etiology and found variants in 15 patients (11%) ( 7 ). Heidet and cols., selecting 377 patients with hyperechogenic multicystic kidney disease or other morphological and functional renal alterations, identified mutations in 75 cases (~20%), of whom 5 (7%) presented with DM ( 8 ). Ulinski and cols. investigated 80 children using only sonographic criteria at the first postnatal examination, including those with a renal phenotype, such as unilateral multicystic dysplasia, uni- or bilateral cystic kidneys, uni- or bilateral renal hypo/dysplasia, and single kidney, and found *HNF1B* variations in 25 patients (31.2%) ( 19 ).

Conversely, in the present series, the majority of cases consisted of ADPKD, a monogenic renal disease with an autosomal dominant inheritance characterized by the progressive development of cysts and considered an important cause of end-stage renal disease. It is worth mentioning that a clinical suspicion of ADPKD, coupled with ultrasound imaging according to criteria of Pei and cols. ( 11 ) and confirmed by family history, is enough to establish the diagnosis of ADPKD with no further need for molecular analysis suggesting either *PKD1* , *PKD2,* or the more rare *GANAB* gene mutations ( 20 , 21 ). However, experimental data have suggested that kidney-specific inactivation of *HNF1B* leads to a polycystic phenotype and that the expression of several genes involved in cystic diseases is severely affected in conditionally inactivated mice, as evidenced by chromatin immunoprecipitation showing that 3 of those genes ( *Umod, Pkhd1* and *Pkd2* ) are directly controlled by *HNF1B* ( 22 ).

That said, we selected our patients based on the presence of renal cysts because this phenotype is one of the most studied in individuals with *HNF1B* gene mutations *.* Cysts have been described in patients with *HNF1B* gene mutations since the earliest descriptions by Bingham and cols. ( 4 ), as well as in several other reports ( 7 , 19 , 23 , 24 ). In 2014, Faguer and cols. proposed a score to improve accuracy in patient selection using several clinical, radiologic, and laboratory phenotypes from patients with suspected variations in *HNF1B.* They analyzed data from 433 patients, including 56 with mutations (13%), and the presence of renal cysts scored high (4 points for each affected kidney) in the identification of such mutations ( 13 ). A cutoff point of 8 was the optimal value, with a sensitivity of 98.2%, a specificity of 41.1%, a positive predictive value (PPV) of 19.8%, and a negative predictive value (NPV) of 99.4% ( 13 ). Furthermore, Clissold and cols. analyzed the performance of the Faguer score in another cohort of 686 patients from the UK (177 positive cases – 25.8%). The authors found that this score discriminated adequately between patients with and without mutations with area under ROC curve of 0.72 but with a lower NPV (85%), as compared to Faguer’s results (99.4%) ( 25 ). Unfortunately, in the Brazilian public health system, where our patients were assisted, data for several phenotypes suggested by Faguer’s score are not routinely available, although in a research context we were able to perform the necessary tests on the sample described here. Even with this important limitation, the score seems to provide good discrimination in our population, taking into account that all positive cases had scores of 8 or higher.

On the other hand, according to Verhave and cols. ( 5 ), due to the functional promiscuity of the *HNF1B* transcription factor involved in the development of many organs, *HNF1B-* associated kidney disease may encompass more than DM and renal cysts.

It is noteworthy that data from Faguer and cols. ( 13 ) was from a younger population than ours (mean age of 17 years), including a large number of children with several other anatomical abnormalities of the urinary tract. Although 11%-30% of the sample had uni- or bilateral cysts, they did not characterize polycystic kidney disease. Additionally, patients with suspected ADPKD without a family history are more likely to have mutations in *HNF1B* ( 5 ). Thus, if we did a sub-analysis of our sample, excluding the majority that already had a defined phenotypic diagnosis of ADPKD (19 patients), our positivity rate could reach 22%. Furthermore, Faguer and cols. suggested that this score should be assessed after ruling out recognizable inherited renal disease such as ADPKD, among others ( 13 ).

Concerning mutation type, the occurrence of whole-gene heterozygous deletions is very frequent, reaching around 50% or more of previously described cases. This whole-gene deletion occurs in the context of a chromosomal microdeletion at 17q12 encompassing 14 genes in addition to *HNF1B* ( 9 , 17 , 18 , 23 , 26 , 27 ). Use of MLPA is necessary because Sanger sequencing is not able to identify this type of mutation. Other types of mutations, such as point mutations or small deletions/insertions, are located primarily in the DNA binding domain ( 13 ). Furthermore, *de novo* mutations are common, found in at least ~50% of cases ( 5 , 7 , 19 ). Our data were similar to those observations, although with a limited number of cases. Our previously published case was a *de novo* heterozygous whole-gene deletion ( 15 ). Of the other 2 cases reported here, the first has a frame shift mutation that is possibly *de novo* because the patient’s parents had no history of DM and renal disease, but this is not possible to define because the parents have passed away and there is no available DNA for testing. The other case had a heterozygous whole gene deletion inherited from his father. In this case, we tested the paternal grandmother, who had no history of DM, and the result was negative. The paternal grandfather had died, so it is not possible to determine if the patient’s father had a *de novo* mutation. Few studies have examined the promoter region of the *HNF1B* gene ( 9 ). In the present study, we analyzed this region, but we did not identify any potentially functional variant.

We chose to recruit patients with hyperglycemia not only within the diabetic range, but also with prediabetes. DM is present in about 45% to 80% of cases and has high specificity in the Faguer score ( 13 ). It is important to take into account that *HNF1B* mutations are not usually associated with DM in childhood, this being a later phenotype ( 6 , 8 ). A French group studied the diabetes phenotype of patients with *HNF1B* mutations in more detail. They analyzed 201 adult patients, 159 of whom had DM. Among them, 40% had a familial history of DM suggesting HNF1B-MODY. The age at diagnosis was > 25 years in 57% of the cases, and 80% had BMI < 25 kg/m^2^. A large portion (80% of cases) had residual pancreatic endocrine function measured by C-peptide even almost 10 years after diagnosis. About 60% of the patients were responsive to the use of sulfonylureas or repaglinide. However, a few years after diagnosis, almost 80% of patients were on insulin therapy for metabolic control. Importantly, the presence of retinopathy and neuropathy was observed in a significant number of cases, and the association of cardiovascular risk factors, such as renal failure, hypertension, and dyslipidemia, was seen in 40% of the cases. In addition, the French researchers noted a genotype-phenotype correlation where those with an entire gene deletion had lower BMI at diagnosis and were more frequently treated with insulin. Interestingly, in this study, the genotype was also associated with different renal function. Compared with point mutations, *HNF1B* deletions were independently associated with normal renal function at DM diagnosis and at follow-up. ( 17 ). Similar results were found in other two previous studies ( 8 , 27 ). Some hypotheses could be raised based on these observations, as other genes affected by whole *HNF1B* gene deletions could also have a role in the clinical differences ( 17 , 28 , 29 ).

Regarding other pancreatic phenotypes, Faguer and cols. demonstrated a high specificity for pancreas hypoplasia and/or exocrine pancreas insufficiency, each phenotype scoring 4 points on Faguer’s scale. Around 11% of their patients presented some of these pancreatic abnormalities ( 13 ). As for our imaging exams, we had 4 patients with agenesis of the pancreatic body and/or tail, including the positive relatives (80%). In the Dubois-Laforgue cohort, 59 individuals (62%) had morphological abnormalities in the pancreas ( 17 ). Clissold and cols. found that, in their cohort, about 4% had pancreatic hypoplasia or exocrine failure. These pancreatic phenotypes were significantly associated with *HNF1B* gene mutations in adults but not in children ( 25 ).

Hypomagnesemia seems to be a good discriminator for *HNF1B* mutations. Van der Made and cols. found *HNF1B* gene mutations in 3 cases with hypomagnesemia as the initial and predominant symptom ( 29 ). In general, the prevalence of hypomagnesemia ranges between 25% and 75% and is slightly lower in pediatric cohorts ( 5 , 13 , 16 , 17 , 29 , 30 ). Adalat and cols. suggested that the *HNF1B* gene is fundamental for the transcription of the *FXYD2* gene, encoding the γ-subunit of the sodium-potassium ATPase expressed in the distal convoluted tubule, which is thought to play a role in transcellular magnesium reabsorption ( 30 ). Furthermore, in the Faguer score, hypomagnesemia scores 2 points ( 13 ). In our study, including the positive relatives, 3 patients had hypomagnesemia (60%)

Our patients, including the positive relatives, also had a high frequency of hypertension and dyslipidemia (80%), whereas in the study by Dubois-Laforgue and cols., 75 (37%) had hypertension and 78 (39%) had dyslipidemia ( 17 ). The reason for these associations is not clear and needs to be investigated further. It could be a bias due to the limited number of studied patients, or it could reflect the usual association between these co-morbidities and DM present in the majority of our cases.

Finally, we would like to point out some limitations of the present study, such as the small sample size, which prevented a more definitive analysis of the real accuracy of our recruitment strategy for *HNF1B* gene positive cases. In addition, we used prediabetes, a mild phenotype of hyperglycemia, as an inclusion criterion. From the total number of cases recruited, 62% (n = 18) would fall into this category. If we had selected only those with DM (n = 10), our positivity rate would have been 20% and may have increased the rate of positive cases.

In conclusion, the frequency of mutations in *HNF1B* gene in Brazilian patients selected for renal cysts and hyperglycemia was 7%. Further studies are needed to optimize patient selection criteria for genetic testing, especially in our population.
